# Advances in the Knowledge of the Molecular Pathogenesis of High-Prevalence Tumors and Its Relevance for Their Future Clinical Management

**DOI:** 10.3390/cancers13236053

**Published:** 2021-12-01

**Authors:** Marta Rodríguez, Ion Cristóbal

**Affiliations:** 1 Pathology Department, IIS-Fundación Jiménez Díaz-UAM, E-28040 Madrid, Spain; 2 Center for the Biomedical Research Network in Oncology (CIBERONC), E-28040 Madrid, Spain; 3 Cancer Unit for Research on Novel Therapeutic Targets, Oncohealth Institute, IIS-Fundación Jiménez Díaz-UAM, E-28040 Madrid, Spain; 4 Translational Oncology Division, Oncohealth Institute, IIS-Fundación Jiménez Díaz-UAM, E-28040 Madrid, Spain

This Special Issue aims to include relevant works that increase our knowledge about the molecular pathways that govern the development and progression of high-prevalence human cancers, which are responsible for most cancer-related deaths worldwide. This is one of the ways to provide oncologists with novel therapeutic tools that can improve the clinical management and outcome of cancer patients. In addition to original articles providing relevant results that will be commented upon in more detail below, this Special Issue also contains several review articles that summarize the current state of the art in crucial aspects of human cancer. Thus, the work by Martinez-Useros et al. [1] reviewed epigenetic pathways and treatments (several under study in current clinical trials) that target epigenetic modifications in highly aggressive tumors. Gundamaraju et al. [2] focused in their manuscript on the molecular mechanisms by which the mitochondria influence cancer biology and their usefulness to develop therapeutic strategies. Another relevant challenge, reviewed by Isaguliants et al. [3], is the increased risk of developed cancer observed in people living with human immunodeficiency virus type 1 (HIV-1), despite a long-term successful implementation of antiretroviral therapy. The authors focused on the oncogenic properties of five viral proteins: envelope protein gp120, accessory protein negative factor Nef, matrix protein p17, transactivator of transcription Tat, and reverse transcriptase RT. All proteins either led to the proliferation of pre-existing malignant cells or induced the malignant transformation of normal cells, which is responsible for the carcinogenic effects of HIV-1. Moreover, Senent et al. [4] reported the importance of the complement system in ovarian cancer, highlighting how certain elements of this system play tumor-promoting roles that decrease the efficacy of distinct therapeutic approaches, and discussing the potential usefulness of the complement as a target of treatments for ovarian cancer. In addition, the work by Nishida [5] reviewed the role of oncogenic signaling pathways on the cancer immunosuppressive microenvironment in hepatocellular carcinoma. Interestingly, this manuscript summarizes the molecular factors that could be determining the efficacy of therapies based on immune checkpoint inhibition. Finally, Guijarro-Hernández and Vizmanos [6] carried out a systematic review summarizing the signaling pathways affected in Ph-negative mieloproliferative neoplasms (MPNs). MPNs are driven not only by a constitutive activation of the JAK2/STAT signaling and JAK2-related pathways, but a complex network of non-canonical pathways that affects key cellular functions such as epigenetic and transcriptional regulation, splicing and additional pathways that confer a highly complex and coordinated program in the tumor cells of these blood disorders.

Regarding the contribution of original articles, our Special Issue contains four pieces of work about breast cancer focused on the identification of molecular aberrations that can serve as novel molecular targets and prognostic markers. Thus, Lee et al. [7] reported that the use of a CD99-derived agonist ligand inhibited EGF-induced EGFR dimerization. This issue involved a PTPN12-dependent c-Src/FAK inactivation that impaired cytoskeletal reorganization and suppressed tumor growth in vivo of the triple negative breast cancer cell line MDA-MB-231. Furthermore, Noblejas-López et al. [8] carried out a genomic mapping that evaluated the presence of alterations in 304 splicing-related genes and their prognostic value in luminal breast cancer patients. They identified that amplifications in *CLNS1A*, *LSM1*, and *ILF2* determined poor outcome. At the functional level, they found that these alterations conferred enhanced proliferation in luminal cell lines that can be pharmacologically reversed by using BET inhibitors. In this line of thinking, an increasing number of publications have shown that the use of BET inhibitors could be a therapeutic approach in triple negative breast cancer (TNBC), and that PP2A is a tumor suppressor that directly targets the bromodomain-containing protein 4 (BRD4) regulating its stabilization and activation. The work of Sanz-Alvarez et al. [9] evaluated the clinical impact of BRD4 phosphorylation levels in TNBC patients. Notably, they observed BRD4 hyperphosphorylation in around 34% of cases, and strongly associated with PP2A inhibition status. Moreover, this alteration was markedly associated with patient recurrence and predicted unfavorable prognosis, suggesting the clinical relevance of the PP2A/BET axis as a potential novel marker in TNBC. Considering these results, and the fact that the PP2A pathway has also been previously reported to be affected in luminal breast cancer, it seems that the PP2A/BET interplay could represent a plausible druggable target to develop alternative therapeutic strategies in certain breast cancer patient subgroups from different molecular subtypes. The same research group also published another study in this Special Issue, in this case about HER2-positive breast cancer models. In their work, Sanz-Alvarez et al. [10] evaluated the efficacy of three different PI3K/AKT/mTOR inhibitors (BEZ235, everolimus, and TAK-228) in a panel of HER2-positive breast cancer cell lines with primary and acquired resistance to Trastuzumab. They found promising results combining TAK-228 with Trastuzumab in all resistant cell lines, observing decreased cell proliferation together with increased apoptosis and G0/G1 cell cycle arrest. Considering these results, the combination of Trastuzumab with PI3K/AKT/mTOR inhibitors emerges as a potential alternative strategy to overcome Trastuzumab resistance in HER2-positive breast cancer.

In the context of prostate cancer, the work by Martínez-Martínez et al. [11] provides novel findings about the role of dual specificity phosphatase 1 (DUSP1). The authors demonstrated that this tumor suppressor leads to Snail downregulation and decreased migration and invasion capabilities of prostate cancer cells through the inhibition of c-Jun N-terminal Kinase (JNK) and extracellular-signal-regulated kinase (ERK). Notably, they also found that the subgroup of prostate cancer patients with an expression pattern DUSP_high_/activated JNKl_ow_/activated ERKl_ow_/Snail_low_ showed better clinical outcome, suggesting its potential utility as molecular marker in this disease. Moreover, Khalil et al. [12] showed relevant results suggesting that the TLK1/NEK1/YAP1 signaling axis plays a key role during the process of androgen-sensitive to androgen-independent conversion, facilitating progression to metastatic castration-resistant prostate cancer. Finally, Papadaki et al. [13] published a comprehensive study in bladder cancer. They found that two secreted extracellular matrix proteins, osteomodulin (OMD), and proline/arginine-rich and leucine repeat protein (PRELP), were selectively expressed in bladder umbrella epithelial cells but markedly downregulated in bladder cancer cells. These two proteins act as tumor suppressors, regulating epithelial to mesenchymal transition (EMT), which was mediated by the inhibition of the TGF-β and EGF pathways.

Altogether, this Special Issue includes several reviews and unique articles with novel, interesting findings that allow the readers to improve their knowledge about the molecular mechanisms involved in high-prevalence tumors and the recent advances in targeted therapies for these diseases.
